# Is Lithium Stabilization a Hidden Parameter in the Chemical Exfoliation of Metallic MoS_2_?

**DOI:** 10.1021/acsami.6c02060

**Published:** 2026-04-21

**Authors:** Mathias Krämer, Yongqiang Kang, J. Manoj Prabhakar, Andrea M. Mingers, Arulkumar Ganapathi, Petra Ebbinghaus, Se-Ho Kim, Yug Joshi, Baptiste Gault

**Affiliations:** † 28272 Max Planck Institute for Sustainable Materials, Max-Planck-Straße 1, Düsseldorf 40237, Germany; ‡ Department of Materials Science and Engineering, 34973 Korea University, Seoul 02841 Republic of Korea; § Univ Rouen Normandie, CNRS, INSA Rouen Normandie, Groupe de Physique des Matériaux, UMR 6634, Rouen F-76000, France

**Keywords:** 2D transition metal dichalcogenides, chemical exfoliation, hydrogen evolution reaction, dopants, atom probe tomography

## Abstract

MoS_2_ nanomaterials have been identified as a cost-effective and earth-abundant alternative to platinum-group metals for electrocatalytic hydrogen evolution. Chemical exfoliation of bulk semiconducting 2H-MoS_2_ by lithium intercalation is widely employed to synthesize metallic 1T-MoS_2_ nanosheets with enhanced basal plane activity and improved charge transport. While the catalytic benefits of this phase transition are well established, the mechanisms responsible for stabilizing the metastable 1T phase following exfoliation remain unexplored. Here, detailed compositional analysis by atom probe tomography uncovers the incorporation of lithium as nanoscale clusters within the 2D material, even after acidic treatment. Operando electrochemical measurements reveal the dissolution of weakly adsorbed lithium upon initial contact with the electrolyte, whereas the lithium clusters seem to remain stable under conditions relevant to the hydrogen evolution reaction. These findings establish detailed compositional analysis as essential for understanding lithium incorporation as a potentially overlooked parameter for controlling and modulating the functional properties and the stability of the metallic 1T phase of chemically exfoliated transition metal dichalcogenides.

## Graphical abstract

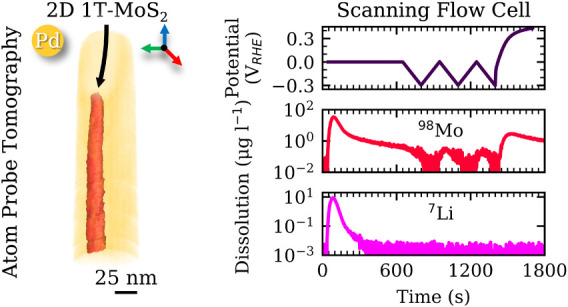

## Introduction

Green hydrogen, as both a clean energy carrier for hard-to-electrify sectors^1^ and as an industrial feedstock for processes such as the sustainable reduction of metal ores^2,3^ has the potential to accelerate global decarbonization toward net zero. Yet water electrolysis, key to large-scale green hydrogen production, is still constrained by the scarcity and high cost of platinum-group metal catalysts. As a result, efforts are focused on screening and designing efficient and robust catalysts from more abundant and affordable materials.^4,5^

2D materials combine an exceptional surface-to-volume ratio with tunable functional properties, making them ideal candidates for surface-active chemical processes, including photocatalysis and electrocatalysis.^6,7^ In the context of water electrolysis, 2D transition metal dichalcogenides (TMDs) and in particular 2D MoS_2_ stands out as affordable and readily available catalyst with activity for the hydrogen evolution reaction (HER).^8–10^ However, the catalytic activity of MoS_2_ nanomaterials in its naturally occurring semiconducting 2H phase is confined to the edge sites.^11,12^ Various approaches have been systematically investigated to overcome the intrinsic inactivity of the MoS_2_ basal plane, including defect engineering via dopants^13,14^ sulfur vacancies^15–17^ and other point defects,^18^ or increasing the density of edge sites.^19–21^ All of these approaches seek to enhance catalytic performance by increasing the density of active sites or by enhancing their intrinsic activity.^22^

Lithium-mediated chemical exfoliation of bulk 2H-MoS_2_ offers a simple yet effective route to activate the catalytic activity of the basal plane, as charge transfer from intercalated lithium drives a local atomic rearrangement that induces a transition to the metastable metallic 1T phase.^23^ In practice, the synthesis of 1T-MoS_2_ nanosheets involves lithium intercalation through organolithium reagents^24^ or through electrochemical methods,^25^ followed by sonication-assisted hydration and exfoliation in polar protic solvents such as water. Although lithium intercalation is crucial for the 2H to 1T phase transition, it is commonly assumed to be fully removed during aqueous exfoliation, yet this has received limited experimental confirmation.

Recent advances in spatially resolved mass spectrometry, including techniques such as atom probe tomography (APT),^26^ have prompted a fundamental reassessment of nanomaterial synthesis.^27^ During synthesis, the (unintentional) incorporation of elements and impurities from the chemicals can influence the functional properties of the as-synthesized nanomaterial.^28^ In different 2D materials, namely MXenes^29^ and 2D transition metal oxides,^30^ the incorporation of elements from the synthesis, particularly alkali metals, has been observed. Given that the intercalation of ions often plays a key role in the top-down synthesis of 2D materials, it is increasingly important to clarify how the synthesis route influences the detailed composition, and thus functional properties.^31^

For chemically exfoliated MoS_2_, the stabilization mechanisms of the metastable metallic 1T phase remain a subject of debate. In this study, 3D compositional mapping at the nanoscale by APT reveals the persistence of lithium clusters within the material after exfoliation in water and subsequent attempted proton-mediated lithium cation exchange. Operando electrochemical dissolution experiments show that, apart from weakly surface-adsorbed lithium, the lithium content remains stable at potentials relevant to the HER. These findings suggest that residual lithium from synthesis plays a crucial role in stabilizing the metastable metallic 1T phase of MoS_2_ under electrocatalytically relevant conditions. Given lithium’s catalytic activity toward the nitrogen reduction reaction,^12^ and its n-type doping effect,^32^ rationalizing and controlling lithium intercalation and adsorption may therefore provide powerful levers for enhancing the functional properties of chemically exfoliated TMDs.

## Results and Discussion

### Synthesis and Structural Characterization

Commercially available bulk 2H-MoS_2_ powder was purchased for the synthesis of metallic 2D MoS_2_, and characterized prior to 2D material synthesis. Scanning electron microscopy (SEM) imaging of the bulk powder shown in Figure 1a and b reveals the characteristic layered morphology of the material, as well as finer, nanosheet-like fragments, likely resulting from mechanical exfoliation during processing. Structural characterization by X-ray diffraction (XRD) in Figure 1c proves that the bulk powder is phase-pure 2H-MoS_2_. All diffraction peaks match the reference pattern (PDF #37–1492; Molybdenite-2H (synthetic), *P*6_3_/*mmc*, *a* = 3.161 Å, *c* = 12.299 Å), with no additional reflections observed, suggesting the absence of crystalline impurities. Complementing the XRD results, X-ray photoelectron spectroscopy (XPS) analysis of the Mo 3*d* core level spectrum in Figure 1d corroborates the presence of 2H-MoS_2_, with no indication of additional oxide phases such as MoO_3_.^33^ However, inductively coupled plasma optical emission spectroscopy (ICP-OES) analysis revealed a nonstoichiometric composition of MoS_1.76_, which may indicate the presence of sulfur vacancies, partial surface oxidation, or both. Analysis of the bulk powder using APT yielded a stoichiometry of MoS_1.56_O_0.17_, confirming the assumption that the precursor material is rich in sulfur vacancies, with a small amount of oxygen incorporated and likely stabilized within these vacancies. The composition was determined by peak decomposition, in which the isotopic ratios are used to deconvolve overlapping peaks in the mass spectrum, including O^+^ and S^2+^, as well as Mo^+++^, $O_{2}^{+}$ , S^+^, and $S_{2}^{++}$ . The deviation in the measured composition between ICP-OES and APT likely reflects a compositional bias, induced by the choice of APT acquisition parameters, specimen geometry, and lack of calibration to a reference.^34^

**Figure 1:**
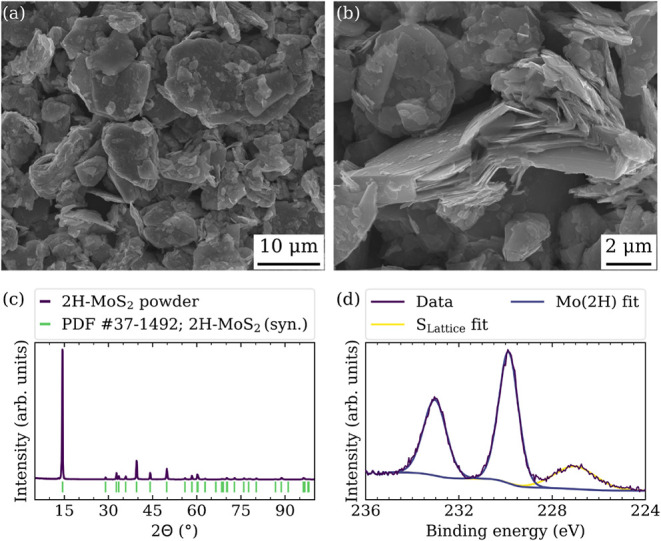
Characterization of the purchased 2H-MoS_2_ precursor powder. (a, b) Electron micrographs of the 2H-MoS_2_ powder. The scanning electron microscopy images were acquired using the secondary electron signal collected with an InLens detector. (c) X-ray diffraction data for the 2H-MoS_2_ powder. (d) High-resolution X-ray photoelectron Mo 3*d* and S 2*s* core level spectrum for the 2H-MoS_2_ powder.

Lithium intercalation with organolithium reagents induces atomic rearrangements in 2H-MoS_2_, resulting in the formation of Li_*x*_MoS_2_. Upon exposure to water, this intermediate readily exfoliates into 1T-MoS_2_ nanosheets, yielding a stable black colloidal ink. For SEM imaging, the as-synthesized material was drop-cast onto a silicon wafer. Figure 2a and b show top-view and cross-sectional SEM images that highlight the morphological change from layered powder particles to an assembly of restacked nanosheets. Raman spectroscopy and XPS analysis confirmed the structural transition from the semiconducting 2H to the metallic 1T phase of MoS_2_. The emergence of the *J*_1_, *J*_2_, and *J*_3_ vibrational modes in the Raman spectrum in Figure 2c, absent in the 2H-MoS_2_ powder, are characteristic for the metallic 1T phase.^35^ Deconvolution of the Mo 3*d* core level spectrum in Figure 2d and the S 2*p* core level spectrum (provided in the Supporting Information) revealed the coexistence of two distinct phases, with the components shifted to lower binding energies relative to 2H-MoS_2_ attributed to 1T-MoS_2_.^33^ The 1T phase fraction was determined to be 86% based on XPS measurements. A stoichiometry of Li_0.21_MoS_1.78_, determined by ICP-OES, indicates incomplete lithium removal during exfoliation and subsequent washing in water, likely due to intercalation or surface adsorption.

**Figure 2:**
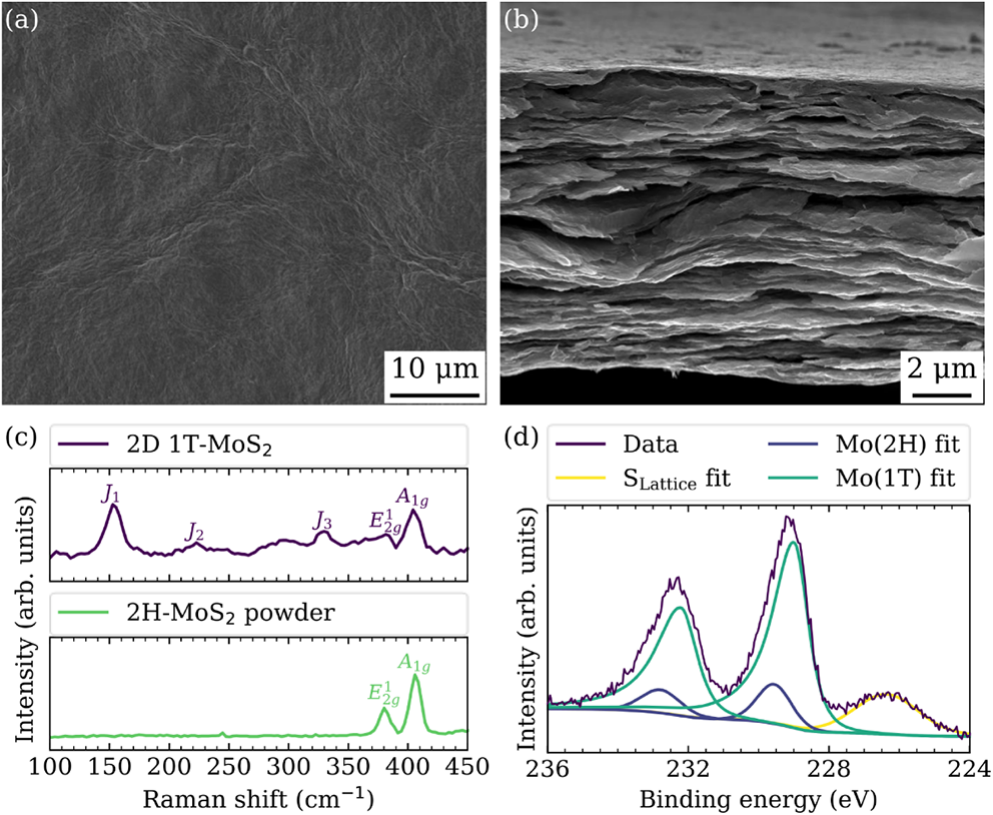
Synthesis of 1T-MoS_2_ nanosheets by chemical exfoliation of bulk 2H-MoS_2_ powder. (a) Electron micrographs of the top and (b) cross-sectional view of a drop-cast 2D 1T-MoS_2_ film. The scanning electron microscopy images were acquired using the secondary electron signal collected with an Everhart-Thornley detector. (c) Raman spectra for the bulk 2H-MoS_2_ powder and the as-synthesized 1T-MoS_2_ nanosheets. (d) High-resolution X-ray photoelectron Mo 3*d* and S 2*s* core level spectrum for the as-synthesized 1T-MoS_2_ nanosheets.

### Stabilization of Lithium in Chemically Exfoliated MoS_2_

Intercalating ions or molecules into 2D materials has become a powerful and versatile strategy for tuning their electronic or catalytic properties.^36^ As lithium intercalation drives the formation of the 1T phase in MoS_2_ along with significant changes in its functional properties^37,38^ it is important to consider whether chemically exfoliated MoS_2_ may contain residual lithium or other impurities from synthesis that stabilize the metastable metallic phase,^39^ similar to what is observed for n-type dopants such as rhenium.^13^ So far, it is assumed that the metallic 1T phase is stabilized by a water bilayer that passivates the excess negative charge on the nanosheets, rather than by residual lithium ions.^40^

To decouple synthesis-induced effects from intrinsic lithium retention, the stacked nanosheets were treated with a Brønsted–Lowry acid to probe proton-mediated lithium exchange. Such a chemical treatment has been demonstrated for MXenes to replace intercalated ions with protons,^41^ or to dissolve crystallized lithium salts.^42^ Drop-cast 1T-MoS_2_ films were immersed in 0.5 M H_2_SO_4_ at room temperature for 24 and 120 h. H_2_SO_4_ was chosen over of other acids such as HCl to ensure consistency with the electrolyte used in subsequent electrochemical measurements.

Following synthesis and acidic treatment of the 2D material films, APT was employed to directly detect and spatially localize lithium via near-atomic-scale 3D compositional mapping. Figure 3a shows the reconstructed 3D atom map of the sample after 120 h of treatment in 0.5 M H_2_SO_4_ (data for the as-synthesized material and the material after 24 h acidic treatment are provided in the Supporting Information). To stabilize the mechanically fragile 2D material specimen during APT analysis, the needle-shaped APT specimen was conformally coated in situ with a thin palladium layer following the protocol described in ref 43. Lithium was detected in the as-synthesized 2D material at (4.04 ± 0.04) at.%, decreasing to (2.54 ± 0.02) at.% after 24 h and to (1.27 ± 0.01) at.% after 120 h of acidic treatment, indicating progressive leaching. Conversely, an increase in oxygen content was observed, rising from (5.74 ± 0.09) at.% in the as-synthesized nanosheets to (18.94 ± 0.04) at.% after 24 h of treatment in 0.5 M H_2_SO_4_, reaching (19.28 ± 0.04) at.% after 120 h, which can be ascribed to oxidation of the 2D material in contact with the electrolyte.^44^

**Figure 3:**
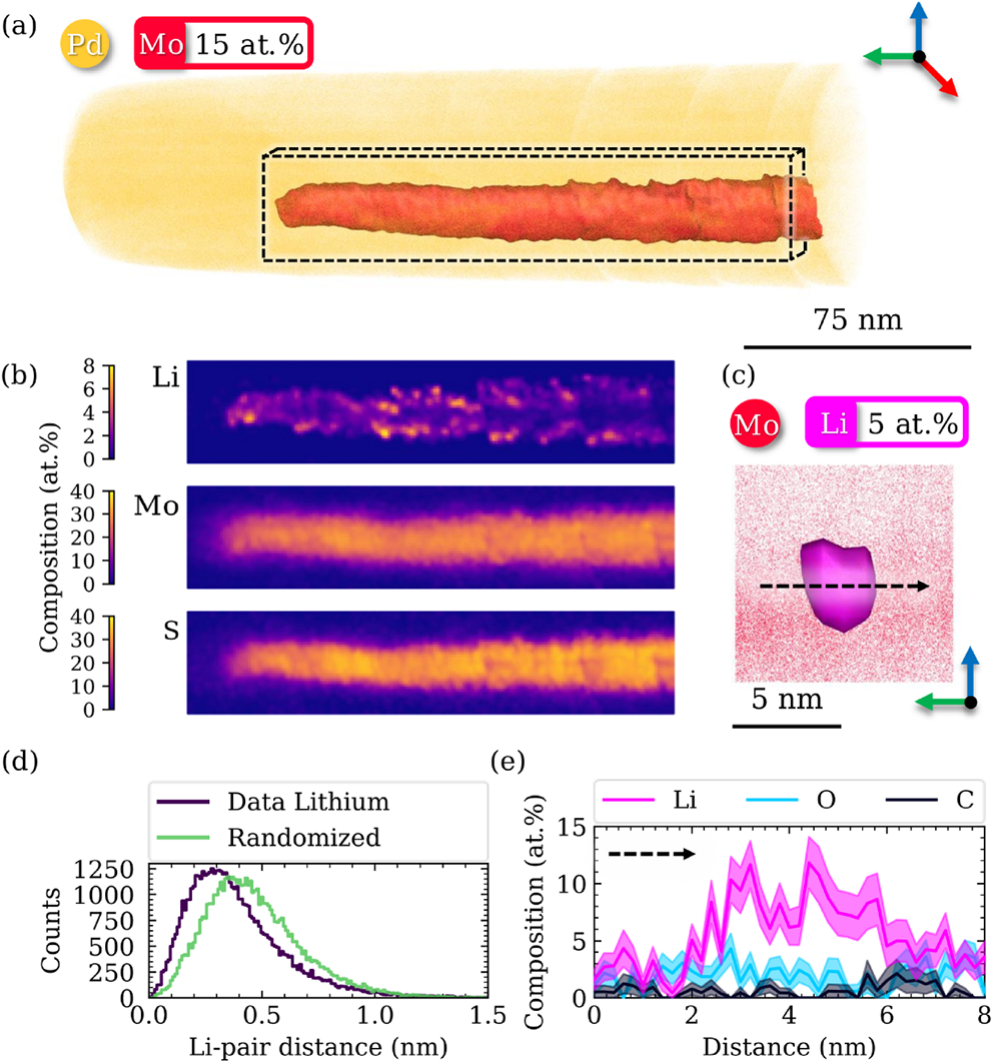
Atom probe analysis of 2D MoS_2_ after 120 h of treatment in 0.5 M H_2_SO_4_ coated in situ with palladium. (a) Reconstructed 3D atom map. (b) 2D compositional contour plots of molybdenum, sulfur, and lithium within the region of interest (190 nm × 40 nm × 5 nm), as indicated in (a). (c) Close-up view of the 3D atom map highlighting a characteristic lithium cluster. (d) First nearest-neighbor analysis for lithium. Sample width ion-pair 0.01 nm. (e) 1D compositional profile (⌀4 nm × 8 nm) across the region of interest as indicated in (c). Errors are estimated according to counting statistics.

The 2D compositional contour plots in Figure 3b resolve the distributions of molybdenum, sulfur, and lithium within the highlighted region of interest. While molybdenum and sulfur are relatively uniformly distributed, lithium exhibits pronounced spatial inhomogeneity, indicative of lithium enriched clusters within the 2D material, with Figure 3c showing such a cluster. A first nearest-neighbor analysis^45^ in Figure 3d further corroborates the observed tendency of lithium toward cluster formation. Comparable behavior has been reported for sodium in 2D MoS_2_,^39^ and potassium in 2D Ti_0.87_O_2_.^30^

Figure 3e provides an example of a 1D compositional profile that reveals no correlation between oxygen or carbon with lithium, which would be indicative of the formation of lithium-containing oxides or lithium carbonate. Lithium may be stabilized either in vacancies,^46^ which would be expected to result in a more homogeneous distribution, or as residual intercalated or surface adsorbed ions. In the latter case, defect-induced localized electric fields are likely to govern their distribution, thereby contributing to the observed clustering.^30^ XPS analysis of the Li 1*s* core level spectrum could provide insight into the chemical nature of these clusters, however, its concentration appears too low to be detected (see Supporting Information for more details).

### Electrochemical Stability under Hydrogen Evolution and Open-Circuit Conditions

Compositional analysis of chemically exfoliated MoS_2_ revealed that a certain amount of lithium is stabilized within the material as clusters, rather than being homogeneously adsorbed on the surface, even after acidic treatment. To investigate the stability of these lithium clusters under HER relevant potentials, a scanning flow cell (SFC) was coupled with inductively coupled plasma–mass spectrometry (ICP-MS) for online analysis of the dissolution.

Element-specific dissolution profiles of molybdenum and lithium toward HER-relevant potentials of the as-synthesized material, obtained from SFC-ICP-MS measurements, are presented in Figure 4. Sulfur was not analyzed due to the use of H_2_SO_4_ as electrolyte, and challenges associated with its detection by ICP-MS.^47^ Following a 600 s potentiostatic hold at 0.0 V vs reversible hydrogen electrode (RHE) to allow the initial electrolyte contact induced dissolution to decay, the electrode was subjected to three cyclic voltammetry (CV) cycles at 2 mV s^–1^ down to −0.3 V vs RHE. After the final cycle down to −0.3 V vs RHE, the sample was maintained at open-circuit potential (OCP) for a further 300 s.

**Figure 4:**
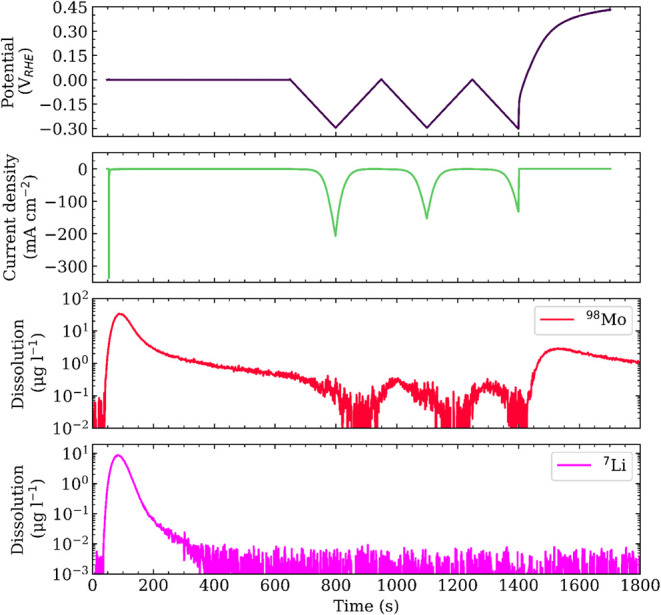
Analysis of the element-specific dissolution profiles of molybdenum and lithium for the as-synthesized 2D 1T-MoS_2_ catalyst as a function of the applied potential, measured using a scanning flow cell setup coupled to an inductively coupled plasma–mass spectrometer.

Within the applied electrochemical protocol, molybdenum remains stable under HER-relevant potentials down to −0.3 V vs RHE. However, toward more anodic potentials, particularly toward OCP conditions, the dissolution increases. A minor temporal delay between dissolution at the working electrode and its detection should be acknowledged. This dissolution behavior is consistent with observations reported in previous studies on MoS_2_-based materials.^44,48^ Conversely, lithium dissolves only initially upon electrolyte contact, after which the dissolution signal rapidly decays into the background, and no further dissolution is detected under either HER-relevant potentials or OCP conditions. APT analysis of a drop-cast 1T-MoS_2_ film on carbon paper (Sigracet 22 BB, SGL Carbon), cycled under identical electrochemical conditions, confirmed residual lithium in the material at a concentration of (2.42 ± 0.03) at.%. Both dissolution profiles are reconfirmed under a modified electrochemical protocol intended to mimic an accelerated stress test (provided in the Supporting Information).

### Outlook on Lithium Stabilization as a Strategy for Functional Property Control

In summary, compositional analysis using APT together with dissolution studies by SFC-ICP-MS demonstrates that chemically exfoliated MoS_2_ contains a weakly adsorbed lithium fraction that can be exchanged upon exposure to a Brønsted–Lowry acid such as H_2_SO_4_. However, a residual amount of lithium remains within the material as nanoscale clusters, presumably intercalated or adsorbed on the surface. This contrasts with the assumption that exfoliation in polar solvents such as water yields a lithium-free material.^40^ Although lithium adsorption can enhance HER performance,^31^ the extent to which lithium influences the reported functional properties of the 2D material remains unclear.

Comparing chemically exfoliated MoS_2_ with materials obtained through other exfoliation methods, such as liquid-phase exfoliation,^49^ is challenging because only lithium intercalation triggers the phase transformation from the semiconducting 2H to the metallic 1T phase. While the 1T phase can also be stabilized by dopants like rhenium, the synthesized materials are either electron beam irradiated, mechanically exfoliated single nanosheets,^50^ or bottom up wet chemically synthesized structures that exhibit morphologies different from those of true 2D materials.^51^

To assess the influence of lithium content on 1T phase stability, the phase fraction was quantified by XPS from the Mo 3*d* core level spectra of the as-synthesized material and the acid-treated samples in Figure 5. To ensure comparability, all samples were prepared from the same batch and investigated with an identical time interval between deposition and measurement. Analysis of the 1T fraction yielded values of 85.1%, 81.7%, and 81.9% for the as-synthesized material and samples treated with 0.5 M H_2_SO_4_ for 24 and 120 h, respectively. The decrease in the 1T phase fraction may be attributed to lithium dissolution upon initial contact with the electrolyte. As the difference between the 24 and 120 h samples lies within the experimental error, no further transition to the semiconducting phase is observed over extended acid treatment. The amount of residual lithium may account for the variation in the 1T phase fraction by stabilizing the metallic phase, though its implications for long-term stability and performance require further investigation.

**Figure 5:**
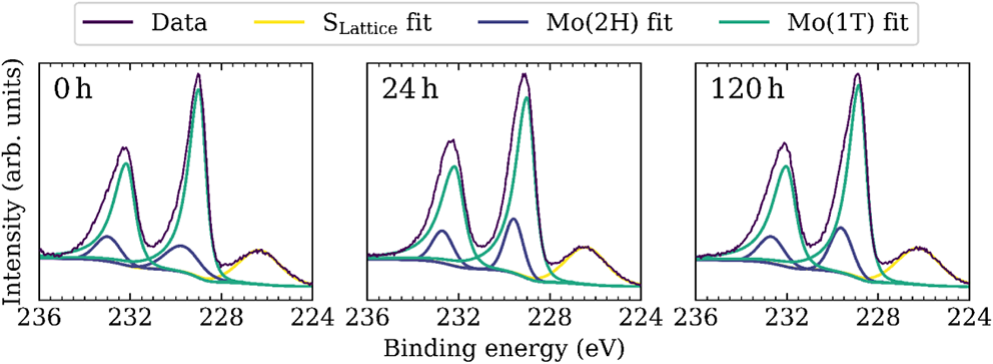
High-resolution X-ray photoelectron Mo 3*d* and S 2*s* core level spectra of the as-synthesized 1T-MoS_2_ nanosheets (0 h) and those treated with 0.5 M H_2_SO_4_ for 24 and 120 h.

Recent studies have demonstrated that careful selection of a polar aprotic exfoliation solvent like tetrahydrofuran can retain intercalated lithium within the exfoliated material, enhancing its performance in applications as sulfur host material in lithium–sulfur batteries by catalyzing the sulfur reduction reaction,^52^ and mitigating polysulfide shuttling through inducing the formation of a quasi-solid-state electrolyte.^53^ Even though the metastable 1T phase can be stabilized at higher temperatures than exfoliated counterparts in polar protic solvents like water, the material is highly susceptible to oxidation under humid conditions.^54^ Lithium intercalation enables not only the direct control of the metastable metallic phase,^55^ but also the precise tuning of its functional properties. Yet it also induces a change in its composition that needs to be acknowledged, particularly to rationalize changes in catalytic activity.^56,57^

Achieving precise control over composition of chemically exfoliated MoS_2_, and potentially 2D TMDs more broadly, requires a detailed mechanistic understanding of lithium stabilization and adsorption. This knowledge is key to the rational design of bulk MoS_2_ precursors and synthesis protocols. Quantification and mapping of residual lithium by APT alone remains insufficient, necessitating atomically resolved techniques such as scanning transmission electron microscopy, which nonetheless suffer from limitations in detecting low concentrations of light elements like lithium.^58,59^ Given that the surface chemistry of non-noble metal electrocatalysts can differ subtly from the assumed as-synthesized material following air exposure or electrolyte contact,^60^ establishing the detailed composition of synthesized catalysts is therefore key to unlocking rational, mechanism-driven electrocatalyst development.^27^

## Conclusion

As the landscape of 2D materials and their various synthesis protocols continues to expand, achieving a precise and comprehensive understanding of their composition has become indispensable. Compositional analysis of chemically exfoliated MoS_2_ demonstrates that lithium nanoclusters persist in the material despite proton-mediated lithium removal. Operando electrochemical investigations employing an SFC-ICP-MS setup indicate that these clusters remain stable under OCP conditions and HER relevant potentials, whereas only weakly adsorbed lithium is dissolved during the initial electrolyte contact. Deliberate retention of lithium appears to be a potential strategy for enhancing the functional properties of 2D TMDs, as lithium may help stabilize the metastable 1T phase. Elucidating how lithium is stabilized within these materials will be key to rationally designing 2D TMDs with enhanced functionality.

## Experimental Methods

### Synthesis of 1T-MoS_2_ Nanosheets by Lithiation and Liquid Exfoliation of Bulk 2H-MoS_2_

Metallic 2D MoS_2_ was synthesized by liquid-phase exfoliation of lithiated bulk MoS_2_. First, 0.4 g of commercially available 2H-MoS_2_ powder (98% purity, 4 μm to 6 μm median particle size; Carl Roth) was immersed in 50 mL of isopropanol and subjected to a precleaning step.^61^ The suspension was sonicated for 1 h in an ice-cooled bath and then centrifuged at 200 × *g* for 1.5 h to remove impurities and small fragments that remain in the supernatant. After centrifugation, the sediment was collected, dried under ambient conditions, and stored overnight in an argon glovebox before lithiation.

For the lithiation of bulk MoS_2_, 4 mL of *n*-butyllithium solution (2.0 M in cyclohexane; Sigma-Aldrich) was added to the precleaned powder under inert argon atmosphere inside the glovebox. The reaction vessel was sealed and transferred out of the glovebox. To promote lithium intercalation,^62^ the mixture was bath sonicated at room temperature for 2 h. After lithiation, excess organolithium reagents were removed by washing the powder three times with 50 mL hexane.

Liquid exfoliation was initiated by dispersing the lithiated powder in deionized water at a ratio of 2 mg mL^–1^, followed by sonication for 1 h in an ice bath. Non-exfoliated material was removed by centrifugation at 500 × *g* for 1.5 h as sediment. The resulting black 1T-MoS_2_ ink was then repeatedly centrifuged at 12300 × *g* and the sediment redispersed in deionized water to remove residual lithium cations.

### Proton-Mediated Lithium Cation Exchange in 1T-MoS_2_

For subsequent proton-mediated lithium cation exchange, thin 2D material films of 1T-MoS_2_ were prepared by drop-casting 5 mL of the as-synthesized colloidal ink onto precleaned pieces of a silicon wafer. After solvent evaporation, the wafers were immersed in 0.5 M H_2_SO_4_ for 24 and 120 h. For the 120 h treatment, the acid solution was refreshed every 24 h.

### Inductively Coupled Plasma Optical Emission Spectroscopy

ICP-OES measurements were performed for both the 2H-MoS_2_ powder and the as-synthesized 1T-MoS_2_ nanosheets using a Thermo Scientific iCAP-6300 Duo instrument. Samples were accurately weighed using a precision balance and then transferred into Teflon digestion vessels, followed by the addition of hydrogen peroxide (H_2_O_2_), nitric acid (HNO_3_), and hydrofluoric acid (HF). The vessels were placed into a microwave digestion system. The temperature was ramped from ambient to 270 °C, and pressure increased to 140 bar over 15 min, with a subsequent holding period of 15 min to ensure complete digestion. Following digestion, the samples were diluted with a 1% HNO_3_ solution to a final volume of 50 mL for elemental analysis.

### Electron Microscopy

Secondary electron imaging was performed with a Zeiss Sigma 500 SEM. The microscope was operated at 5 kV and 1.4 nA with a working distance of 5 mm.

### X-ray Diffraction

XRD analysis of the 2H-MoS_2_ powder was carried out using a Rigaku SmartLab 9 kW system, equipped with Cu Kα radiation (λ = 1.54059 Å). The XRD spectrum was acquired with a step size of 0.01° in the 2Θ range from 5° to 100° for the 2H-MoS_2_ powder.

### X-ray Photoelectron Spectroscopy

XPS spectra were acquired with a Physical Electronics PHI Quantera II spectrometer using an Al–Kα source at 1486.6 eV. Pass energy was set at 55 eV to record the core level spectra of molybdenum (Mo 3*d*) and lithium (Li 1*s*), and at 26 eV to record the core level spectra of sulfur (S 2*p*). The survey scan spectrum was obtained with a pass energy of 140 eV. All the measurements were recorded using a takeoff angle of 45°. Energy step size of 0.025 eV was used for the high-resolution core level spectra, and 0.25 eV was used to collect the survey scan spectrum.

Peak fitting of the core level spectra obtained during the XPS studies was performed using CasaXPS software version 2.3.25. A Shirley-type background was used for all the analyzed spectra. All symmetric photoemission components, namely the S 2*s* and 2*p* peaks and the semiconducting 2H-MoS_2_ contributions in the Mo 3*d* core level, were fitted using a Gaussian (70%)–Lorentzian (30%) peak shape. The metallic 1T-MoS_2_ contributions in the Mo 3*d* core level were fitted using a Lorentzian asymmetric peak shape of LA (α = 1.1, β = 2.3, *m* = 2). Binding energies were calibrated by referencing the adventitious carbon core level signal (C 1*s*) at 284.8 eV.

### Raman Spectroscopy

Raman spectroscopy was conducted using a WITec alpha300 confocal Raman microscope, operated with a green solid-state laser (λ = 532 nm), operated at a laser power of approximately 100 μW. A 50× magnification objective lens and a 600 lines mm^–1^ diffraction grating were used. Raman spectra were recorded with 10 accumulations of 10 s for the bulk 2H-MoS_2_ powder, and with fewer accumulations (3) but longer acquisition times of 60 s for the 1T-MoS_2_ nanosheets. To analyze the 2D material, the aqueous material ink was drop-cast onto a microscope slide.

### Atom Probe Tomography

Needle-shaped APT specimens of the 2D material films were prepared using a dual beam scanning electron microscope—gallium focused ion beam instrument (Helios Nanolab 600, FEI) following the workflow developed and optimized for 2D material films by Krämer et al.^30,43^ By sputtering a thin conformal metallic coating onto the APT specimen, mechanically fragile specimens can be stabilized^63,64^ and the preferential migration of alkali metals under the intense electrostatic field during analysis can be effectively suppressed.^65,66^ Palladium (EVOCHEM Advanced Materials, 99.95% purity) was chosen as sputtering material to minimize oxygen incorporation during the coating procedure.^30^ Palladium sputtering was performed at 30 kV and 48 pA for 45 s to 60 s, repeated 4 times with the specimen rotated by 90° between each sputter cycle. Due to their susceptibility to premature fracture, APT specimens prepared from the bulk 2H-MoS_2_ powder were also coated with palladium.

APT analyses were performed using a 5000 XR (reflectron-fitted) local electrode atom probe (Cameca Instruments), operating in ultraviolet (λ = 355 nm) laser-pulsing mode. Parameters were set to a base temperature of 60 K, a laser pulse energy of 70 pJ, a laser pulsing rate of 125 kHz, and a target detection rate of 5 ions per 1000 pulses on average.

Data reconstruction and analysis were done with AP Suite 6.3 by Cameca Instruments following the default voltage-based reconstruction algorithm.

### Scanning Flow Cell Measurements Coupled with Inductively Coupled Plasma–Mass Spectrometry

Operando electrochemical elemental dissolution was quantified using an SFC connected to a potentiostat (Reference 600, Gamry Instruments) and an ICP-MS (NexION 300X, PerkinElmer). The SFC features 1.9 mm V-shaped channels, with the electrolyte outlet coupled to the ICP-MS. A platinum wire (0.5 mm diameter, 99.997%, Alfa Aesar) positioned in the inlet channel served as the counter electrode, while an Ag/AgCl (3 M KCl) electrode placed in the outlet channel functioned as the reference electrode. The electrolyte solution of 0.1 M H_2_SO_4_ was prepared from suprapure H_2_SO_4_ (96%, Merck) and deionized water (PureLab Flex2, Elga, 18 MΩ cm^–1^, total organic carbon <3 ppb), and pumped with a flow rate of approximately 380 μL min^–1^ through the cell. All measured potentials were referenced to the RHE according to the Nernst equation:

$$V_{\mathrm{RHE}}=V_{\mathrm{Ag}/\mathrm{AgCl}}+0.211\;V+0.059\times \mathrm{pH}$$

To ensure precise calibration of potentials on the RHE scale, daily pH measurements of the electrolyte were carried out using a MultiLab 540 pH meter.

The catalyst ink for the working electrode was prepared by thoroughly mixing 5 mg of the as-synthesized 1T-MoS_2_ nanosheets in a solution of 50 μL Nafion, 900 μL deionized water, and 300 μL ethanol. Following sonication, 1 μL of the catalyst ink was drop-cast onto a polished glassy carbon electrode and left to dry overnight. The working electrode was then positioned beneath the SFC such that the deposited area was enclosed by the cell opening.

A four-point calibration of the ICP-MS was performed every day prior to the measurements and used to convert the detected intensities to the concentration of the dissolved ions in the electrolyte (^7^Li and ^98^Mo). Sc and Rh served as internal standards for Li and Mo, respectively, and were added to the electrolyte via a Y-connector placed downstream of the SFC to correct for physical interferences.

The electrochemical protocol started with a 600 s potentiostatic hold at 0.0 V vs RHE while the sample was brought into contact with the electrolyte under potential control. Three CV scans were performed at a scan rate of 2 mV s^–1^ between 0.0 V and −0.3 V vs RHE. The final CV scan was interrupted upon reaching −0.3 V, after which the sample was held under OCP conditions for additional 300 s.

## Supplementary Material



## Data Availability

The data that support the findings of this study are available from the corresponding authors upon reasonable request.

## Notes

APT characterization of the bulk 2H-MoS2 precursor powder; APT characterization of the 2D material (as-synthesized, after 24 h H_2_SO_4_ treatment, and after electrochemical testing); X-ray photoelectron spectroscopy survey scans, high-resolution S 2*p* core level spectra for both 2H-MoS_2_ powder and 1T-MoS_2_ nanosheets, high-resolution Li 2*p* core level spectra for H_2_SO_4_-treated samples; extended results from the scanning flow cell dissolution experiments (PDF)
