# Supplementary material for: Is Lithium Stabilization a Hidden Parameter in the Chemical Exfoliation of Metallic MoS2?
Source: ACS Appl Mater Interfaces. 2026 Apr 21;18(17):24951–8. doi: 10.1021/acsami.6c02060 (PMC13154132; doi:10.1021/acsami.6c02060)
Supplement: Supplementary file 1 [file am6c02060_si_001.pdf]

Supporting Information
Is Lithium Stabilisation a Hidden Parameter in the Chemical Exfoliation of Metallic MoS_2?

Mathias Krämer [1,*], Yongqiang Kang [1], J. Manoj Prabhakar [1], Andrea M. Mingers[1], Arulkumar Ganapathi [1], Petra Ebbinghaus[1], Se-Ho Kim [2], Yug Joshi [1], and Baptiste Gault [1,3,*]

[1]Max Planck Institute for Sustainable Materials, Max-Planck-Straße 1, 40237 Düsseldorf, Germany
[2]Department of Materials Science and Engineering, Korea University, Seoul, 02841 Republic of Korea
[3]Univ Rouen Normandie, CNRS, INSA Rouen Normandie, Groupe de Physique des Matériaux, UMR 6634, F-76000 Rouen, France
[*]Corresponding authors: m.kraemer@mpi-susmat.de, baptiste.gault1@univ-rouen.fr

Atom Probe Tomography

Compositional Analysis of the Bulk 2H-MoS_2 Powder

The composition of the bulk 2H-MoS_2 powder was characterised using inductively coupled plasma optical emission spectroscopy and atom probe tomography (APT) before chemical exfoliation. Figure S1 (a) shows a exemplary reconstructed 3D atom map, and Figure S1 (b) shows the corresponding 1D compositional profile through the material of interest. Because APT specimens prepared from bulk 2H-MoS_2 powder particles were highly susceptible to premature fracture, in situ palladium coating was applied to enhance mechanical stability, following the same workflow used for the 2D material films.[1,2]

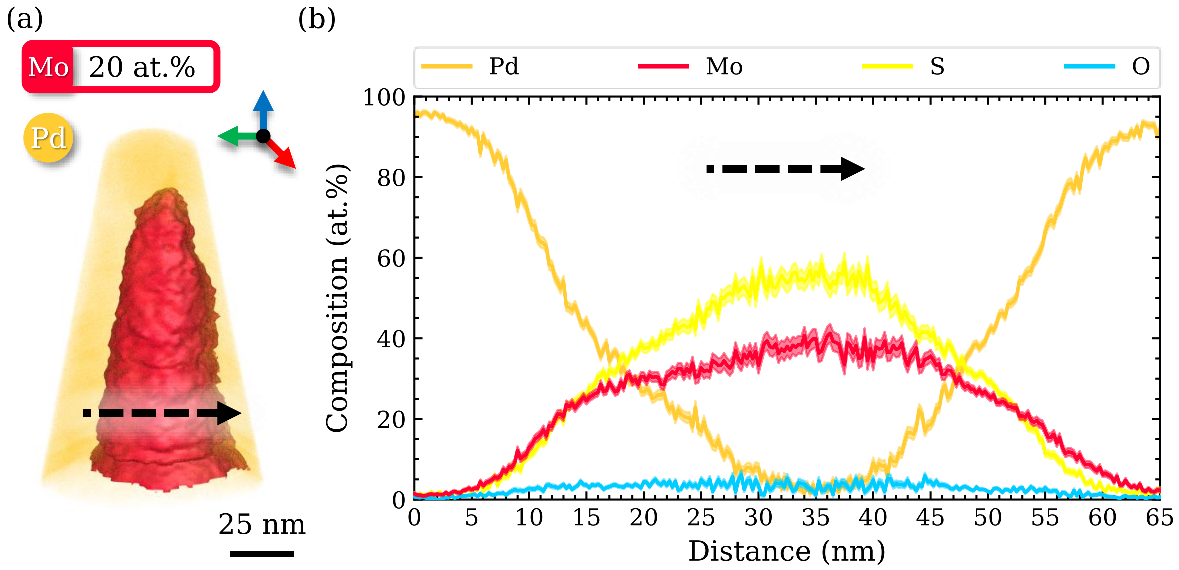

Figure S1: Atom probe analysis of the bulk 2H-MoS_2 powder coated in situ with palladium. (a) Reconstructed 3D atom map. (b) 1D compositional profile (⌀15 nm x 65 nm) across the region of interest as indicated in (a). Errors are estimated according to counting statistics.

Table S1 summarises the bulk composition of the 2H-MoS_2 powder as determined by APT analysis. A cylindrical analysis volume with dimensions of ⌀25 nm x 50 nm was positioned at the centre of the region of interest shown in Figure S1 (a) to minimize reconstruction artifacts, including intermixing zones caused by trajectory aberrations and local magnification effects arising from differences in the evaporation fields of the elements.[3] In this analysis volume, a peak decomposition was performed to deconvolve overlapping peaks in the mass spectrum based on isotopic ratios. The detected carbon

could either stem from the in situ palladium coating,[2] or be present as an impurity in the powder itself, as reported in the manufacturer's data sheet.

Table S1: Bulk composition of the 2H-MoS_2 powder as determined by atom probe analysis.

Mo	S	O	C
36.51 ± 0.13	57.08 ± 0.28	6.39 ± 0.12	0.02 ± 0.01

Data given in at.%.

Mass Spectrum Analysis of Chemically Exfoliated 1T-MoS_2 Nanosheets

An exemplary APT mass spectrum of the palladium coated 2D 1T-MoS_2 is provided in Figure S2, with the identified peaks summarized in Table S2.

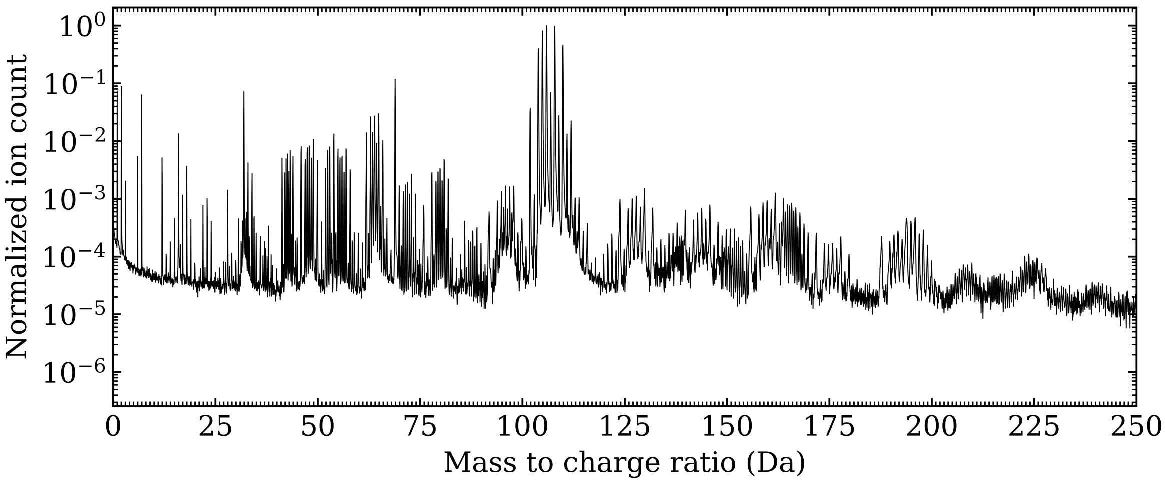

Figure S2: Exemplary mass spectrum of the 1T-MoS_2 nanosheets coated in situ with palladium. Bin width is 0.05 Da

Table S2: List of identified peaks in the mass spectrum of the 1T-MoS_2 nanosheets coated in situ with palladium. Despite the detection of hydrogen-related peaks, no quantification of the hydrogen content was performed.

Mass to Charge Ratio (Da)	Assigned Ion
1	H^+
2	$H_2{}^+$
3	$H_3{}^+$
6	Li^+, C^+
7	Li^+
12	C^+
13	C^+
14	$CH_2{}^+$
15	$CH_3{}^+$
16	S^{++}, O^+

Continued on next page

Table S2 (continued)

Mass to charge ratio (Da)	Assigned Ion
16.5	S^{++}
17	S^{++}, OH^{+}
18	S^{++}, $OH_2{}^{+}$
19	$OH_3{}^{+}$
24	$C_2{}^{+}$
28	CO^{+}
30.7	Mo^{+++}
31.3	Mo^{+++}
31.7	Mo^{+++}
32	Mo^{+++}, S^{+}, $O_2{}^{+}$, $S_2{}^{++}$
32.3	Mo^{+++}
32.5	S^{++}
32.7	Mo^{+++}
33	S^{+}, $O_2{}^{+}$, $S_2{}^{++}$
33.3	Mo^{+++}
33.5	$S_2{}^{++}$
34	S^{+}, $O_2{}^{+}$, $S_2{}^{++}$
34.5	$^{69}Ga^{++}$
36	MoO^{+++}
36.7	MoO^{+++}
37	MoO^{+++}
37.3	MoO^{+++}
37.7	MoO^{+++}
38	MoO^{+++}
38.7	MoO^{+++}
41.3	MoS^{+++}
41.7	MoS^{+++}
42	MoS^{+++}
42.3	MoS^{+++}
42.7	MoS^{+++}
43	MoS^{+++}
43.3	MoS^{+++}

Continued on next page

Table S2 (continued)

Mass to charge ratio (Da)	Assigned Ion
43.7	MoS^{+++}
44	MoS^{+++}, $CO_2{}^{+}$
44.3	MoS^{+++}
44.7	MoS^{+++}
46	Mo^{++}
47	Mo^{++}
47.5	Mo^{++}
48	Mo^{++}
48.5	Mo^{++}
49	Mo^{++}
50	Mo^{++}
51	Pd^{++}
52	Pd^{++}
52.5	Pd^{++}
53	Pd^{++}
54	Pd^{++}, MoO^{++}
55	Pd^{++}, MoO^{++}
55.5	MoO^{++}
56	MoO^{++}
56.5	MoO^{++}
57	MoO^{++}
57.5	MoO^{++}
58	MoO^{++}
59	MoO^{++}, PdO^{++}
60	PdO^{++}
60.5	PdO^{++}
61	PdO^{++}
62	MoS^{++}, PdO^{++}
62.5	MoS^{++}
63	MoS^{++}, PdO^{++}
63.5	MoS^{++}
64	MoS^{++}, $S_2{}^{+}$

Continued on next page

Table S2 (continued)

Mass to charge ratio (Da)	Assigned Ion
64.5	MoS^{++}
65	MoS^{++}, $S_2{}^+$
65.5	MoS^{++}
66	MoS^{++}, $S_2{}^+$
66.5	MoS^{++}
67	MoS^{++}, $S_2{}^+$
68	MoS^{++}, $S_2{}^+$
69	$^{69}Ga^+$
70	$MoSO^{++}$
71	$MoSO^{++}$
71.5	$MoSO^{++}$
72	$MoSO^{++}$
72.5	$MoSO^{++}$
73	$MoSO^{++}$
73.5	$MoSO^{++}$
74	$MoSO^{++}$
74.5	$MoSO^{++}$
75	$MoSO^{++}$
78	$MoS_2{}^{++}$
78.5	$MoS_2{}^{++}$
79	$MoS_2{}^{++}$
79.5	$MoS_2{}^{++}$
80	$MoS_2{}^{++}$
80.5	$MoS_2{}^{++}$
81	$MoS_2{}^{++}$
81.5	$MoS_2{}^{++}$
82	$MoS_2{}^{++}$
82.5	$MoS_2{}^{++}$
83	$MoS_2{}^{++}$
84	$MoS_2{}^{++}$
86	MoS_2O^{++}
86.5	MoS_2O^{++}

Continued on next page

Table S2 (continued)

Mass to charge ratio (Da)	Assigned Ion
87	MoS_2O^{++}
87.5	MoS_2O^{++}
88	MoS_2O^{++}
88.5	MoS_2O^{++}
89	MoS_2O^{++}
89.5	MoS_2O^{++}
90	MoS_2O^{++}
90.5	MoS_2O^{++}
91	MoS_2O^{++}
92	Mo^+, MoS_2O^{++}
94	Mo^+, ${MoS_3}^{++}$
94.5	${MoS_3}^{++}$
95	Mo^+, ${MoS_3}^{++}$
95.5	${MoS_3}^{++}$
96	Mo^+, ${MoS_3}^{++}$
96.5	${MoS_3}^{++}$
97	Mo^+, ${MoS_3}^{++}$
97.5	${MoS_3}^{++}$
98	Mo^+, ${MoS_3}^{++}$
98.5	${MoS_3}^{++}$
99	${MoS_3}^{++}$
99.5	${MoS_3}^{++}$
100	Mo^+, ${MoS_3}^{++}$
102	Pd^+
103	PdH^+
104	Pd^+, ${PdH_2}^+$
105	Pd^+, PdH^+, ${PdH_2}^+$
106	Pd^+, PdH^+, ${PdH_2}^+$
107	PdH^+, ${PdH_2}^+$
108	Pd^+, PdH^+, ${PdH_2}^+$, MoO^+
109	PdH^+, ${PdH_2}^+$
110	Pd^+, PdH^+, ${PdH_2}^+$, MoO^+

Continued on next page

Table S2 (continued)

Mass to charge ratio (Da)	Assigned Ion
111	PdH^+, $PdH_2{}^+$, MoO^+
112	PdH^+, $PdH_2{}^+$, MoO^+
113	$PdH_2{}^+$, MoO^+
114	MoO^+
115	MoO^+
116	MoO^+
118	PdO^+, MoO^+
119	$PdOH^+$
120	PdO^+
121	PdO^+, $PdOH^+$
122	PdO^+, $PdOH^+$
123	$PdOH^+$
124	MoS^+, PdO^+
125	MoS^+, $PdOH^+$
126	MoS^+, PdO^+
127	MoS^+, $PdOH^+$
128	MoS^+
129	MoS^+
130	MoS^+, $PdOC^+$
131	MoS^+
132	MoS^+, $PdOC^+$
133	MoS^+, $PdOC^+$
134	MoS^+, $PdOC^+$
135	$PdOC^+$
136	$PdOC^+$
137	$PdOC^+$
138	$PdOC^+$
139	$PdOC^+$
140	$MoSO^+$
141	$MoSO^+$
142	$MoSO^+$
143	$MoSO^+$

Continued on next page

Table S2 (continued)

Mass to charge ratio (Da)	Assigned Ion
144	$MoSO^+$
145	$MoSO^+$
146	$MoSO^+$
147	$MoSO^+$
148	$MoSO^+$
149	$MoSO^+$
150	$MoSO^+$
156	${MoS_2}^+$
157	${MoS_2}^+$
158	${MoS_2}^+$
159	${MoS_2}^+$
160	${MoS_2}^+$
161	${MoS_2}^+$, Pd_3C^{++}
162	${MoS_2}^+$, Pd_3C^{++}
162.5	Pd_3C^{++}
163	${MoS_2}^+$, Pd_3C^{++}
163.5	Pd_3C^{++}
164	${MoS_2}^+$, Pd_3C^{++}
164.5	Pd_3C^{++}
165	${MoS_2}^+$, Pd_3C^{++}
165.5	Pd_3C^{++}
166	${MoS_2}^+$, Pd_3C^{++}
166.5	Pd_3C^{++}
167	Pd_3C^{++}
167.5	Pd_3C^{++}
168	Pd_3C^{++}
169	Pd_3C^{++}
169.5	Pd_3C^{++}
170	Pd_3C^{++}
170.5	Pd_3C^{++}
171	Pd_3C^{++}
172	MoS_2O^+

Continued on next page

Table S2 (continued)

Mass to charge ratio (Da)	Assigned Ion
173	MoS_2O^+
174	MoS_2O^+
175	MoS_2O^+
176	MoS_2O^+
177	MoS_2O^+
178	MoS_2O^+
179	MoS_2O^+
180	MoS_2O^+
181	MoS_2O^+
182	MoS_2O^+
188	$MoS_3{}^+$
189	$MoS_3{}^+$
190	$MoS_3{}^+$
191	$MoS_3{}^+$
192	$MoS_3{}^+$
193	$MoS_3{}^+$
194	$MoS_3{}^+$
195	$MoS_3{}^+$
196	$MoS_3{}^+$
197	$MoS_3{}^+$
198	$MoS_3{}^+$
199	$MoS_3{}^+$
200	$MoS_3{}^+$
204	MoS_3O^+
205	MoS_3O^+
206	MoS_3O^+
207	MoS_3O^+
208	MoS_3O^+
209	MoS_3O^+
210	MoS_3O^+
211	MoS_3O^+
212	MoS_3O^+

Continued on next page

Table S2 (continued)

Mass to charge ratio (Da)	Assigned Ion
213	MoS_3O^+
214	MoS_3O^+
220	$MoS_4{}^+$
221	$MoS_4{}^+$
222	$MoS_4{}^+$
223	$MoS_4{}^+$
224	$MoS_4{}^+$
225	$MoS_4{}^+$
226	$MoS_4{}^+$
227	$MoS_4{}^+$
228	$MoS_4{}^+$
229	$MoS_4{}^+$
230	$MoS_4{}^+$
232	$MoS_4{}^+$

Characterisation of the 2D material (as-synthesised, after 24 h H_2SO_4 treatment, and after electrochemical testing)

Figure S3, Figure S4, and Figure S5 summarize the atom probe analyses of as-synthesized 1T-MoS_2, following 24 h H_2SO_4 treatment, and after electrochemical testing. In each figure, subfigure (a) shows the reconstructed 3D atom map, (b) displays the 2D compositional contour plots, and (c) presents the first nearest-neighbour analysis. All datasets indicate a tendency for lithium clustering.

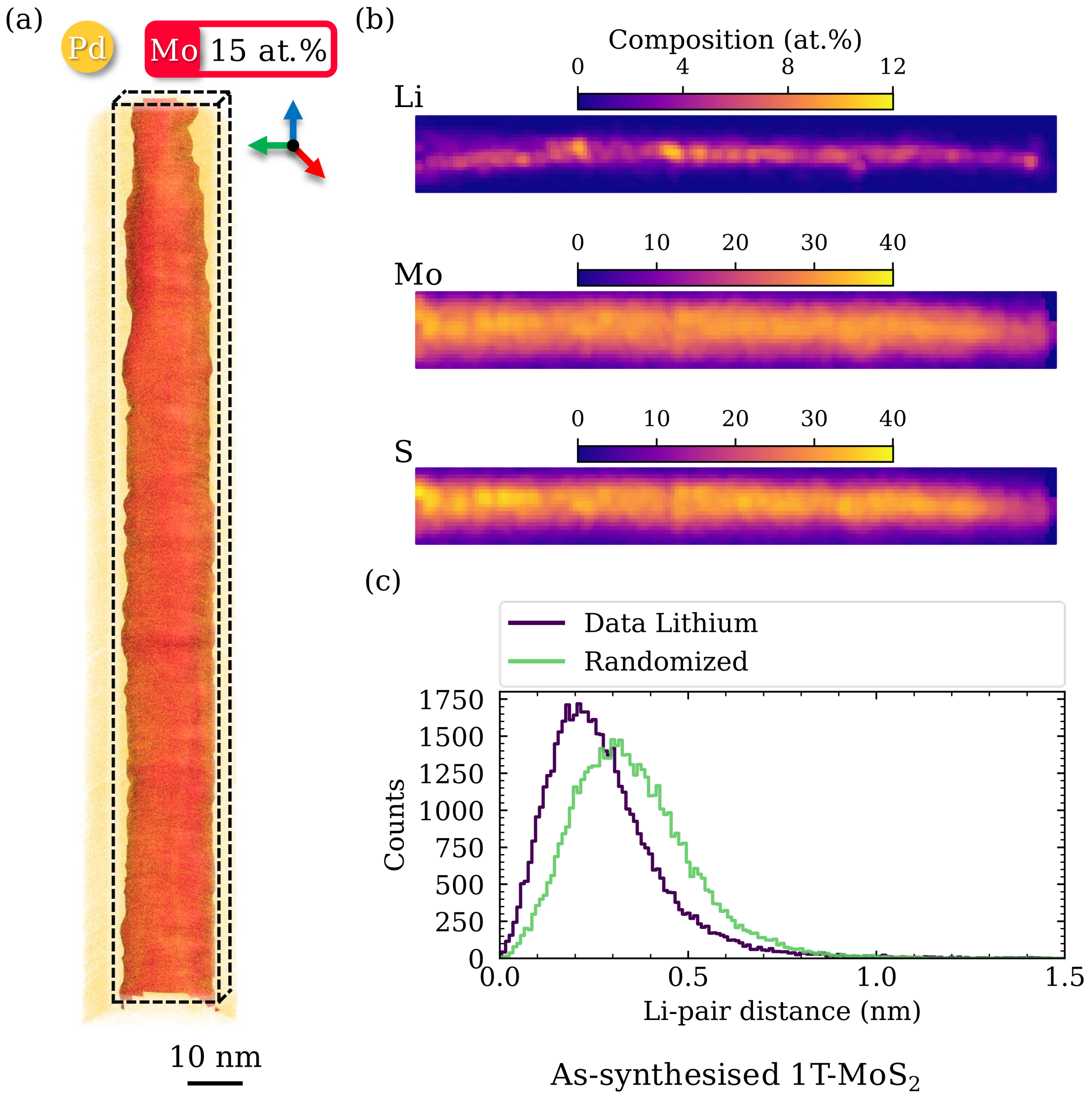

Figure S3: Atom probe analysis of the as-synthesised 2D MoS_2 nanosheets coated in situ with palladium. (a) Reconstructed 3D atom map. (b) 2D compositional contour plots of molybdenum, sulphur, and lithium within the region of interest (170 nm x 20 nm x 5 nm), as indicated in (a). (c) First nearest-neighbour analysis for lithium. Sample width ion-pair 0.01 nm.

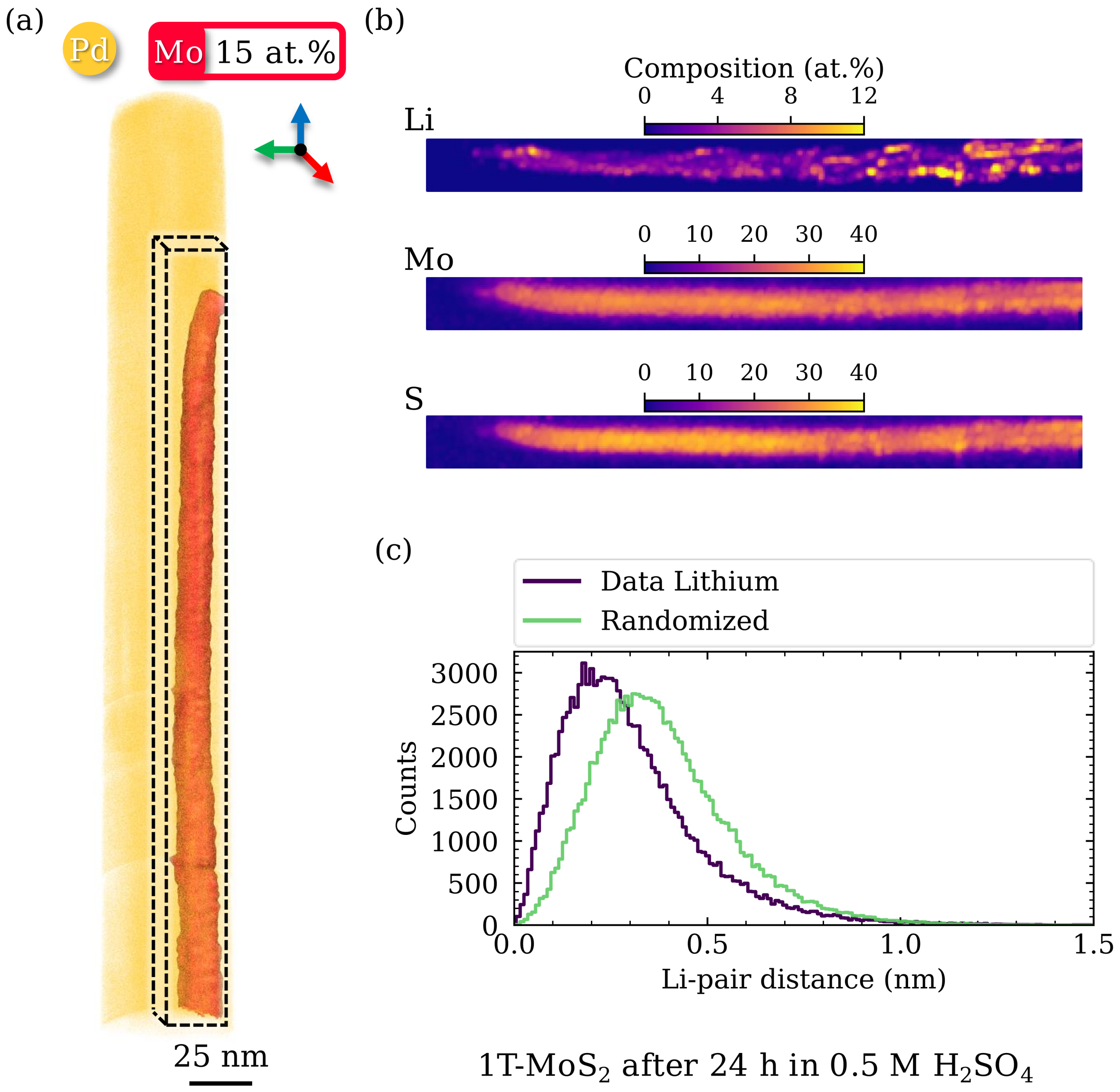

Figure S4: Atom probe analysis of 2D MoS_2 after 24 h of treatment in 0.5 M H_2SO_4 coated in situ with palladium. (a) Reconstructed 3D atom map. (b) 2D compositional contour plots of molybdenum, sulphur, and lithium within the region of interest (320 nm x 25 nm x 5 nm), as indicated in (a). (c) First nearest-neighbour analysis for lithium. Sample width ion-pair 0.01 nm.

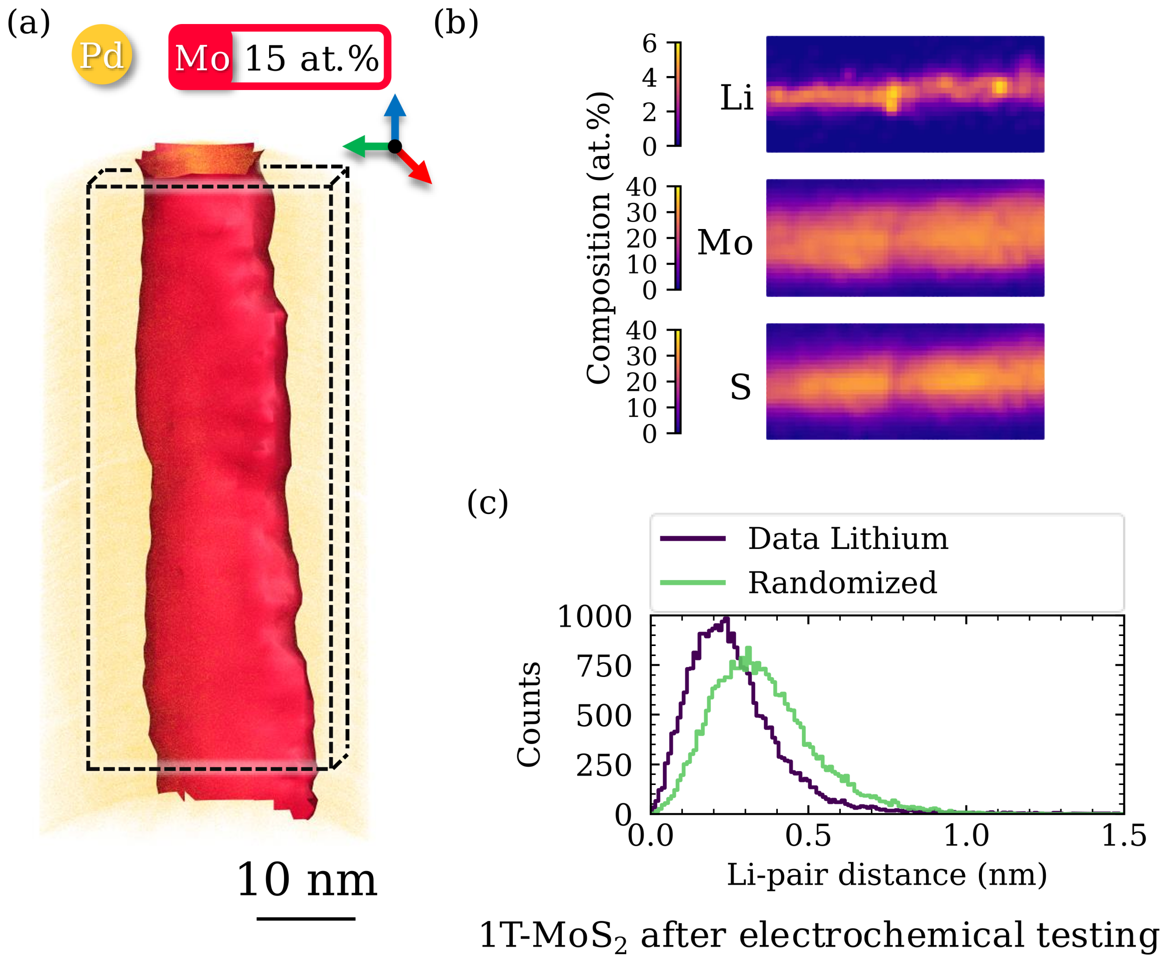

Figure S5: Atom probe analysis of 2D MoS_2 after electrochemical testing coated in situ with palladium. (a) Reconstructed 3D atom map. (b) 2D compositional contour plots of molybdenum, sulphur, and lithium within the region of interest (60 nm x 25 nm x 5 nm), as indicated in (a). (c) First nearest-neighbour analysis for lithium. Sample width ion-pair 0.01 nm.

X-ray Photoelectron Spectroscopy

A complete X-ray photoelectron spectroscopy survey spectrum with all assigned core-level signals is shown in Figure S6 and Figure S7 for the bulk 2H-MoS_2 powder and the chemically exfoliated 1T-MoS_2 nanosheets, respectively.

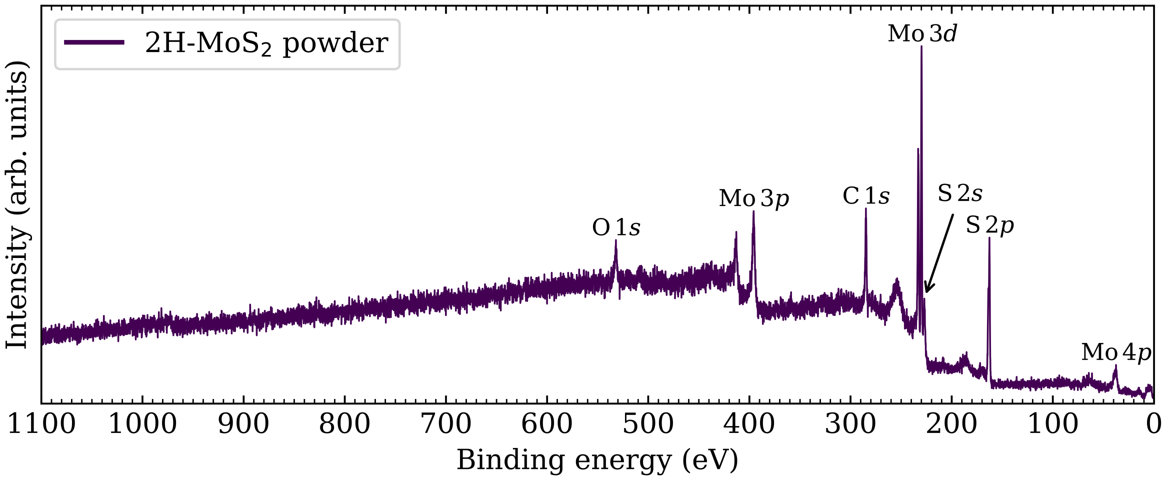

Figure S6: X-ray photoelectron spectroscopy survey spectrum of the bulk 2H-MoS_2 powder.

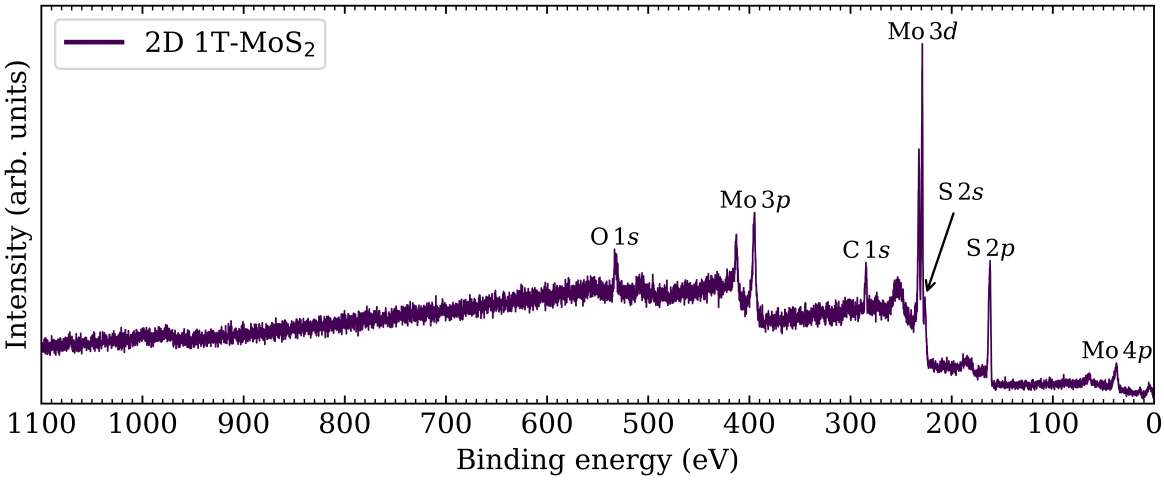

Figure S7: X-ray photoelectron spectroscopy survey spectrum of the as-synthesised 1T-MoS_2 nanosheets.

Additional high-resolution S 3*d* core level spectra for both materials are provided in Figure S8. As observed for the Mo 3*d* core level spectrum of the chemically exfoliated 2D material, the spectrum can be deconvolved into two distinct phases. The additional features are shifted towards lower binding energies relative to the semiconducting 2H phase, indicating the presence of the metallic 1T phase.

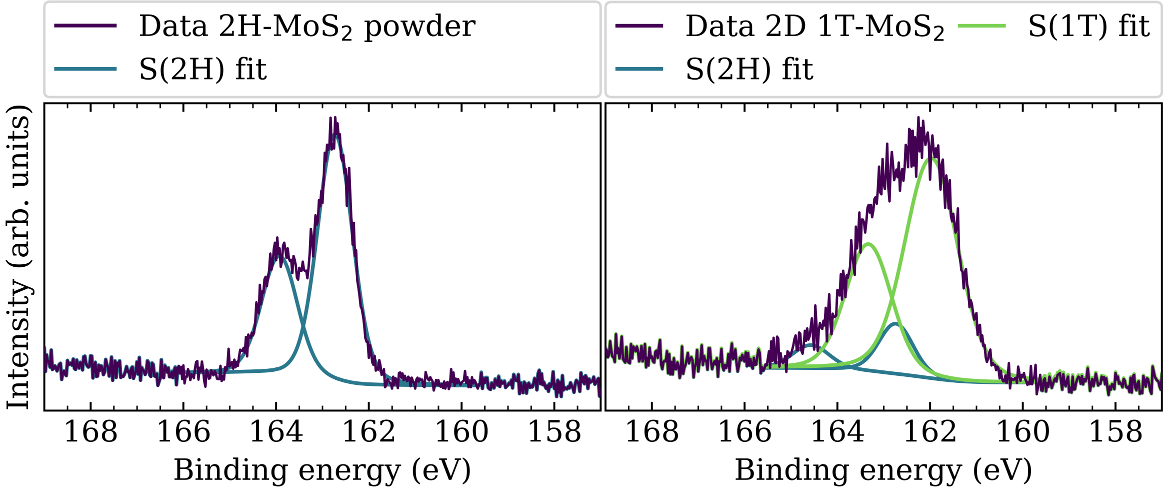

Figure S8: High-resolution X-ray photoelectron S 2*p* core level spectra for the bulk 2H-MoS_2 powder and the as-synthesised 1T-MoS_2 nanosheets.

No Li $2p$ signal between 55 eV and 57 eV was detected in the as-synthesised or H_2SO_4-treated samples (Figure S9), which could help elucidate the chemical nature of the lithium clusters. This may be attributed to the low lithium concentration in the samples and the challenges associated with detecting lithium in materials containing significantly heavier elements.[4]

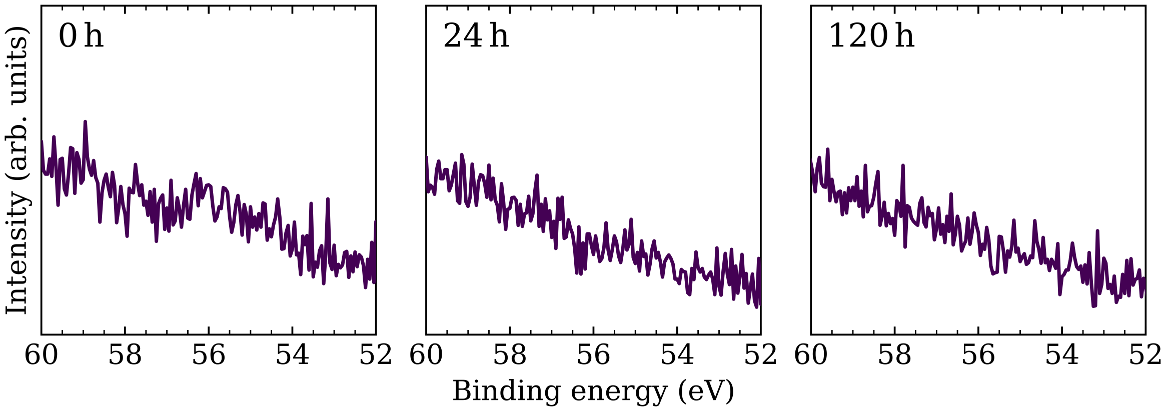

Figure S9: High-resolution X-ray photoelectron Li $1s$ core level spectra of the as-synthesised 1T-MoS_2 nanosheets (0 h) and those treated with 0.5 M H_2SO_4 for 24 h and 120 h.

Scanning Flow Cell Measurements coupled with Inductively Coupled Plasma - Mass Spectrometry

A second electrochemical protocol was employed to mimic an accelerated stress test of the as-synthesised 2D catalyst using the scanning flow cell setup coupled to an inductively coupled plasma - mass spectrometer for online analysis of the dissolution. Figure S10 summarises the results, which corroborate findings and conclusions presented in the manuscript. The second electrochemical protocol also started with a 600 s potentiostatic hold at 0.0 V vs. reversible hydrogen electrode (RHE) while the sample was brought into contact with the electrolyte under potential control, to allow the initial electrolyte contact induced dissolution to decay. Cyclic voltammetry (CV) was performed at varying scan rates between 0.0 V and −0.3 V vs. RHE. Three initial CV scans were performed at $10\,\mathrm{mV\,s^{-1}}$, followed by 50 CV scans at $200\,\mathrm{mV\,s^{-1}}$, and three final CV scans at $10\,\mathrm{mV\,s^{-1}}$. A 100 s rest interval at 0.0 V vs. RHE was applied between CV scans conducted at different scan rates.

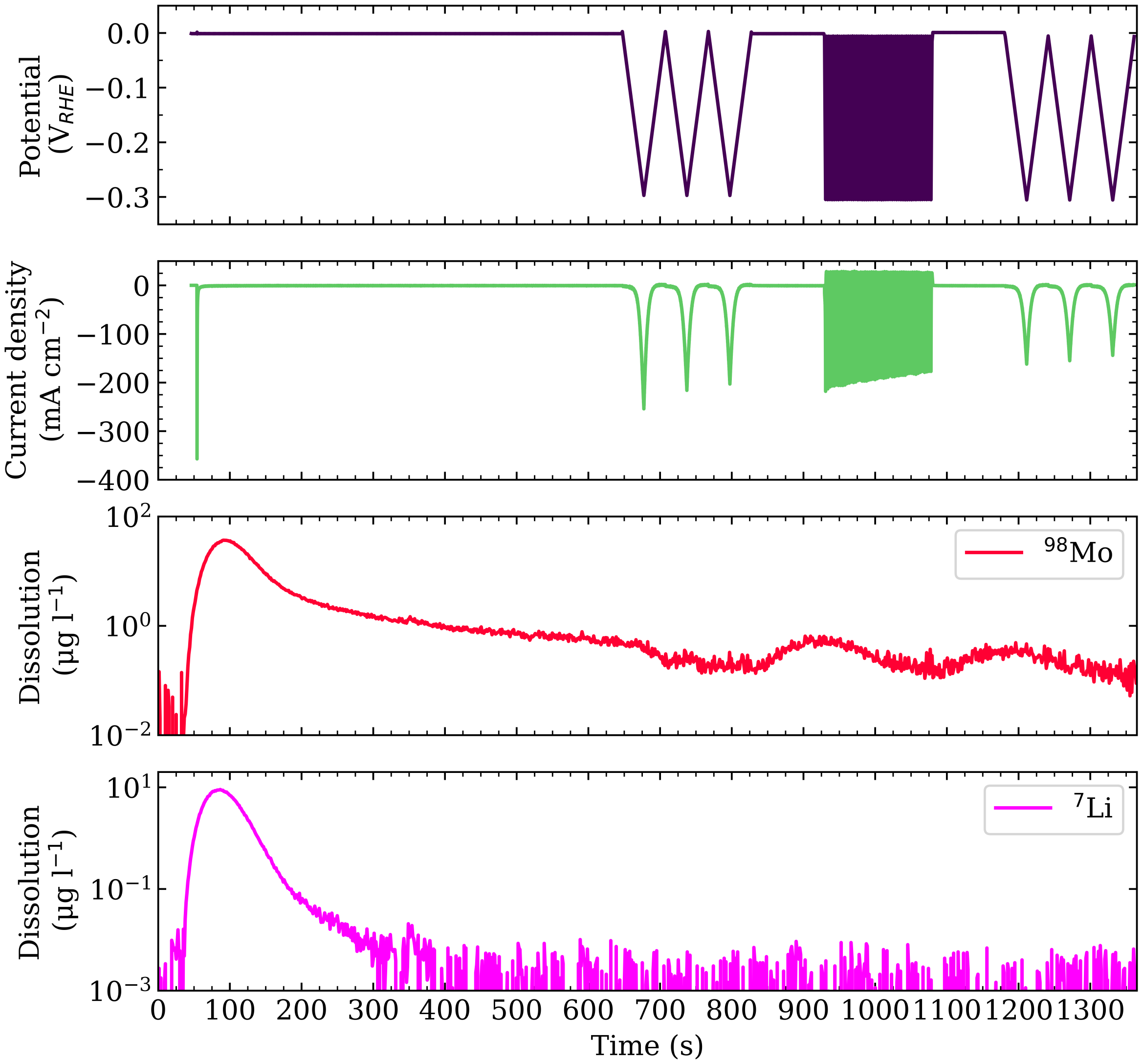

Figure S10: Analysis of the element-specific dissolution profiles of molybdenum and lithium for the as-synthesised 2D 1T-MoS_2 catalyst as function of the applied potential, measured using a scanning flow cell setup coupled to an inductively coupled plasma - mass spectrometer.

The cyclic voltammograms in Figure S11, which are replotted from the data in Figure S10, show a significant increase in the cathodic current at negative potentials, indicating electrocatalytic activity toward the hydrogen evolution reaction. However, at higher scan rates, a hysteresis becomes apparent, suggesting additional interfacial and capacitive contributions.

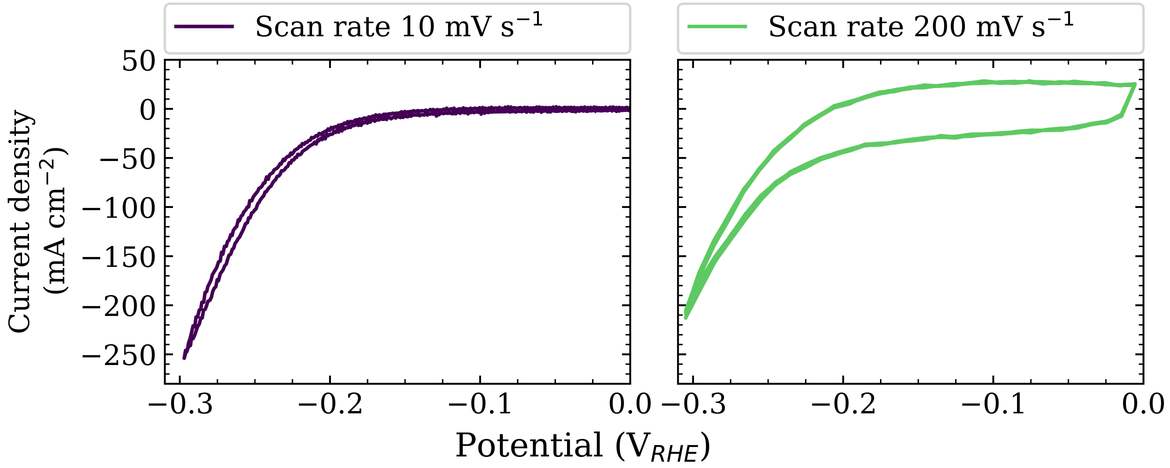

Figure S11: Cyclic voltammograms of the 2D 1T-MoS_2 catalyst at varying scan rates.

References

(1) Krämer, M.; Favelukis, B.; Sokol, M.; Rosen, B. A.; Eliaz, N.; Kim, S.-H.; Gault, B. Facilitating Atom Probe Tomography of 2D MXene Films by In Situ Sputtering. *Microscopy and Microanalysis* **2024**, *30*, 1057–1065, DOI: 10.1093/mam/ozae035.

(2) Krämer, M.; Favelukis, B.; Prabhakar, J. M.; Albrecht, A.; Rosen, B. A.; Eliaz, N.; Sokol, M.; Gault, B. Compositional complexity in a 2D transition metal oxide. *Materials Today Nano* **2026**, *34*, 100790, DOI: 10.1016/j.mtnano.2026.100790.

(3) Miller, M. K. The effects of local magnification and trajectory aberrations on atom probe analysis. *J. Phys. Colloques* **1987**, *48*, C6-565-C6–570, DOI: 10.1051/jphyscol:1987692.

(4) Shard, A. G. Detection limits in XPS for more than 6000 binary systems using Al and Mg Kα X-rays. *Surface and Interface Analysis* **2014**, *46*, 175–185, DOI: 10.1002/sia.5406.
